# Identity, proliferation capacity, genomic stability and novel senescence markers of mesenchymal stem cells isolated from low volume of human bone marrow

**DOI:** 10.18632/oncotarget.7456

**Published:** 2016-02-17

**Authors:** Gabrielis Kundrotas, Evelina Gasperskaja, Grazina Slapsyte, Zivile Gudleviciene, Jan Krasko, Ausra Stumbryte, Regina Liudkeviciene

**Affiliations:** ^1^ Department of Botany and Genetics, Faculty of Natural Sciences, Vilnius University, Vilnius, Lithuania; ^2^ Biobank, National Cancer Institute, Vilnius, Lithuania; ^3^ Laboratory of Immunology, National Cancer Institute, Vilnius, Lithuania

**Keywords:** human mesenchymal stem cells, genomic stability, long-term expansion, PCR arrays, senescence markers, Gerotarget

## Abstract

Human bone marrow mesenchymal stem cells (hBM-MSCs) hold promise for treating incurable diseases and repairing of damaged tissues. However, hBM-MSCs face the disadvantages of painful invasive isolation and limited cell numbers. In this study we assessed characteristics of MSCs isolated from residual human bone marrow transplantation material and expanded to clinically relevant numbers at passages 3-4 and 6-7. Results indicated that early passage hBM-MSCs are genomically stable and retain identity and high proliferation capacity. Despite the chromosomal stability, the cells became senescent at late passages, paralleling the slower proliferation, altered morphology and immunophenotype. By qRT-PCR array profiling, we revealed 13 genes and 33 miRNAs significantly differentially expressed in late passage cells, among which 8 genes and 30 miRNAs emerged as potential novel biomarkers of hBM-MSC aging. Functional analysis of genes with altered expression showed strong association with biological processes causing cellular senescence. Altogether, this study revives hBM as convenient source for cellular therapy. Potential novel markers provide new details for better understanding the hBM-MSC senescence mechanisms, contributing to basic science, facilitating the development of cellular therapy quality control, and providing new clues for human disease processes since senescence phenotype of the hematological patient hBM-MSCs only very recently has been revealed.

## INTRODUCTION

Human mesenchymal stem cells (hMSCs) are non-hematopoietic, adherent fibroblast-like cells with intrinsic ability of self-renewal and potential for multilineage differentiation [1]. The stromal compartment of bone marrow (BM) was the first biological material from which MSCs were isolated. Since then, BM-derived MSCs have been the most widely studied and are thought to be key regulators of BM physiology [2].

MSCs are the major stem cells for cell therapy and have been used in the clinic for approximately 10 years [3]. Currently, BM represents the major source of MSCs for clinical use [4]. Stem cell-based therapy using human BM-MSCs (hBM-MSCs) holds promise for treating degenerative diseases, cancer, and repair of damaged tissues, where limited therapeutic options exist [5]. E.g., Wernicke et al. reported a high (73.8%) overall response to MSC therapy of the life-threatening severe steroid-refractory graft *versus* host disease [6]. Disadvantage of using hBM-MSCs is the limited cell numbers obtained from invasive isolation techniques [7]. This has led many researchers to investigate alternate sources of human MSCs, including adipose tissue [8] and umbilical cord [9], that can be used in the clinical setting.

High quantities of MSCs are needed for clinical applications, thus requiring extensive cell expansion in long-term culture [10]. However, the occurrence of karyotypic instability in cultured hBM-MSCs has been documented. It has been admitted that genome instability enables tumor cells to acquire their characteristics [11], therefore the tumorigenesis potential of the hMSCs has become the most important concern for clinical use of MSCs [12]. Though, hBM-MSC studies presented highly conflicting results. It has been shown that hBM-MSCs *in vitro* acquire chromosomal aberrations, undergo spontaneous transformation and form tumors *in vivo* [13]. In contrast, other groups have documented normal karyotype throughout hBM-MSC culture and no malignant transformation *in vivo* [14, 15]. Besides, it has been shown that hBM-MSCs do not transform spontaneously *in vitro* and chromosomal instability occurs without leading to malignant transformation, possibly being only a sign of cell senescence [16]. Cellular senescence, which refers to irreversible cell growth arrest [17], is another issue related to hBM-MSC cultivation. It limits the proliferative capacity of primary cells in culture [18], impairs therapeutic potential of hBM-MSC [19], and increases the risk of cell neoplastic transformation [20, 21]. Although some publications reporting the alarming finding of malignant transformation of hMSCs [22], including hBM-MSCs [23], later on have been retracted [24, 25], there is still debate concerning the genetic stability of hMSCs and the implication for clinical safety [26, 27].

It is of great scientific interest to investigate MSCs isolated from low human bone marrow volume for potential medical use. Recently, our group has showed that MSCs can be successfully isolated by red blood cell lysis method from residual bone marrow transplantation material and expanded *in vitro* to clinically relevant numbers [28]. The aim of this study was therefore to assess hBM-MSC immunophenotype as proposed by The International Society for Cellular Therapy (ISCT) [29]; to evaluate proliferative capacity, senescence status and cytogenetic stability, as determined by The European Medicine Agency (EMA) [30]; and to apply array technology as suggested by the U.S. Food and Drug Administration (FDA) [31]. Our results highlight the identity, proliferation capacity, and genomic safety of MSCs isolated from low human bone marrow volume and reveal 38 new hBM-MSCs potential senescence markers during prolonged cultivation *in vitro*.

## RESULTS

### Morphology

**Figure 1:**
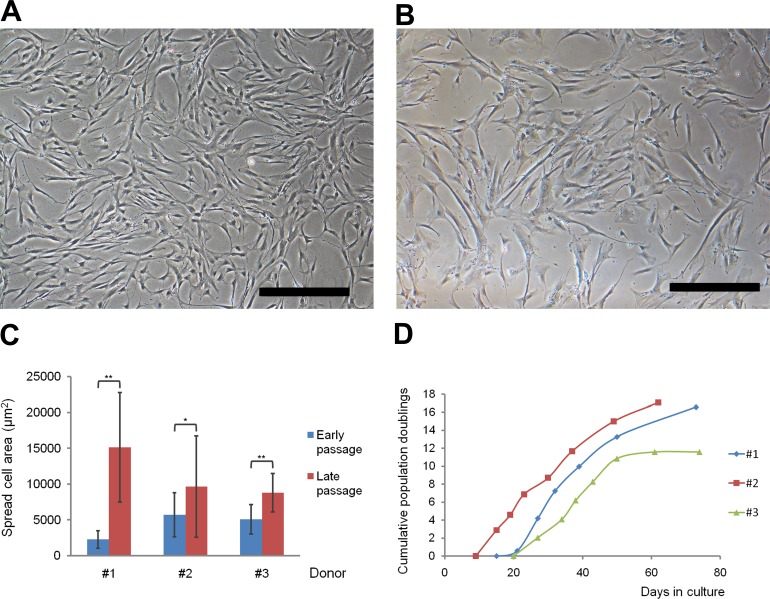
Morphology and proliferation kinetics of hBM-MSCs during *in vitro* culture Typical homogeneous population of fibroblast-like cells at P4 **A.** and heterogenous population including enlarged cells with altered morphology at P7 **B.** Original magnification x40, scale bars represent 500 μm. **C.** Average spread cell area at early (P3-P4) and late (P6-P7) passages of individual samples (*P < 0.05; **P < 0.001). **D.** Growth kinetics of MSC cultures from 3 donors scored as cumulative population doublings (y-axis) plotted against time in culture (x-axis). Each marker represents a passage.

We observed hBM-MSCs microscopically at every passage. Adherent long spindle-shaped or flat fibroblast-like cells were detected 24-48 hours after isolation. Such morphology retained up to passages 3-4 (P3-P4) (Figure 1A). Later on the proportion of enlarged cells with altered morphology gradually increased, which became obvious at late passages 6-7 (P6-P7) (Figure 1B). An average spread cell area was significantly enlarged at late passages of individual samples (Figure 1C).

### Proliferation

MSCs showed a slower proliferation after isolation and reached P1 after 21±6 days (Figure 1D). From P1 to P3 (sample #1) or P4 (samples #2 and #3) the cells proliferated faster and CPDs resulted in 8.08±0.74 after additional 14.00±2.65 days. In late culture the gradual slow-down in the cellular growth occurred and it took 34.67±5.51 more days to complete with 15.08±3.04 CPDs.

### Flow cytometry analysis

**Figure 2:**
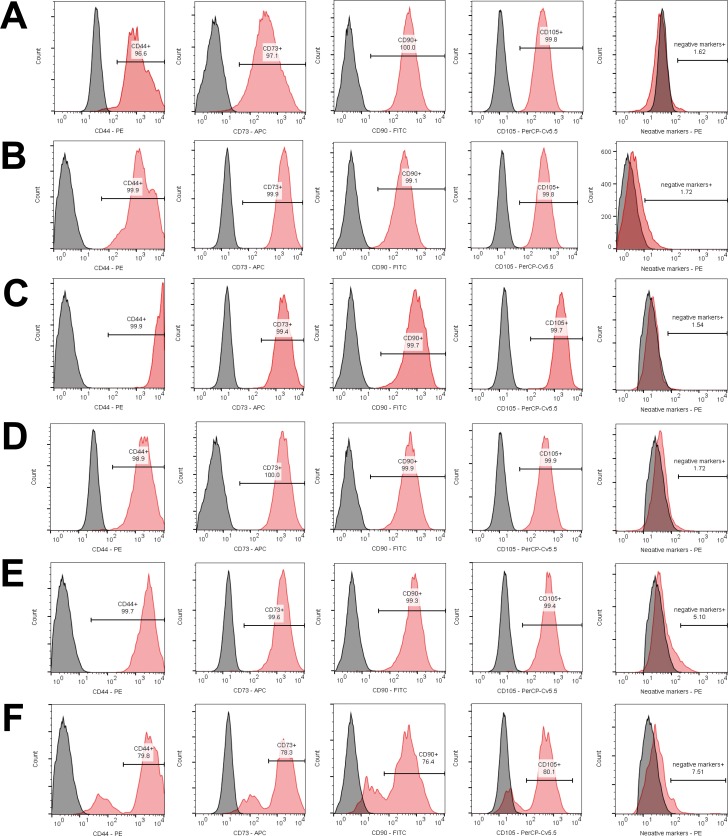
Immunophenotype of hBM-MSCs in long term culture Analysis of samples #1 **A.**, #2 **B.**, #3 **C.** at the early passages and analysis of samples #1 **D.**, #2 **E.**, #3 **F.** at the late passages is demonstrated. Histograms on the left (grey) represent unstained cells, and histograms on the right (red) represent stained cells.

In the early passages over 99% of the cells were positive for CD73, CD90, and CD105, while below 2% of the cells expressed CD11b, CD19, CD45, CD34, and HLA-DR (Figure 2A-2C). However, part of MSC population of #3 sample lost the expression of positive markers and gained the expression of negative markers in P7 (Figure 2F). The expression of negative markers also increased in #2 sample in P7, although expression of positive markers remained stable (Figure 2E). The immunophenotype of #1 sample in P6 did not change (Figure 2D). Mean viability of hBM-MSCs was 94.02±2.92% at early passages and 93.47±5.61% at late passages. The side-scatter (SSC) was 337.00±55.44 units at early passages and 391.67±27.00 units at late passages, although the difference was not statistically significant (*P* = 0.085).

### Senescence-associated β-galactosidase staining

**Figure 3:**
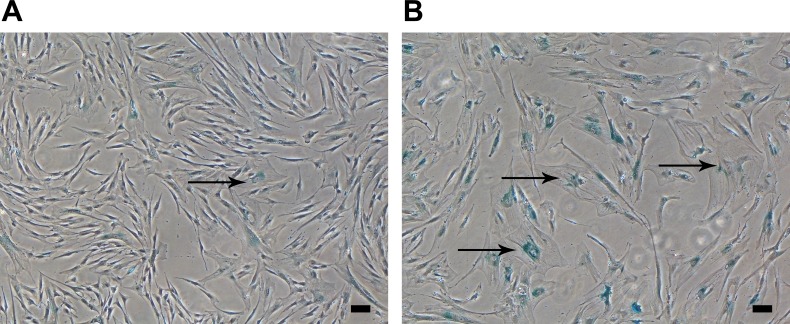
hBM-MSC senescence during *in vitro* culture Representative images of enlarged with altered morphology SA-β-gal-positive cells (indicated with black arrows) at P4 **A.** and P7 **B.** Original magnification x40, scale bars represent 100 μm.

The cell dyeing for SA-β-gal showed that long-term culture is accompanied by increase in senescent cells. There were 1.59±0.94% SA-β-gal-positive cells at P3-P4 and 41.97±4.57% at P6-P7 (*P* = 0.0043) (Figure 3A-3B).

### Karyotype

**Figure 4:**
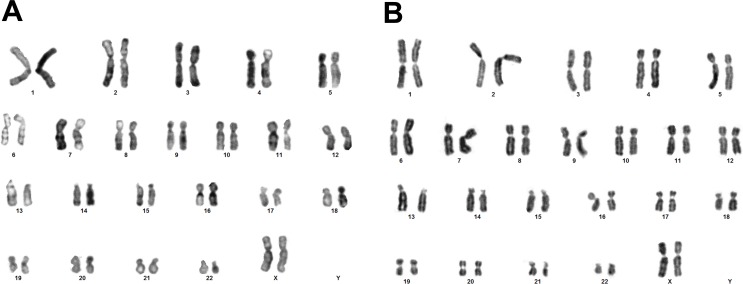
hBM-MSC karyotype during *in vitro* culture Representative karyograms of hBM-MSC normal diploid karyotype (2n, *n* = 23) at P3 **A.** and P6 **B.**

To investigate the effects of long-term culture on genomic integrity, we analyzed the karyotype by G-banding at P3-P4 and P6-P7 (Figure 4A-4B). Nearly 87% of the cells at early passages and nearly 88% of the cells at late passages had normal diploid karyotype (2n, *n* = 23). No clonal numerical or structural cytogenetic alterations were observed. We detected random aneuploidies at early passages 45,-10; 47,+15; 47,+22 (sample #1); 44,-20,-21 and 45,-22 (sample #2); and 47,+22 (sample #3). At late passages we detected 44,-X,-20 (sample #1) and 45,-20 (sample #2) (data not shown). None of these abnormalities was considered clonal because they all were seen in a single cell per specimen, most likely, due to technical preparation of chromosomes.

### Gene expression

**Figure 5:**
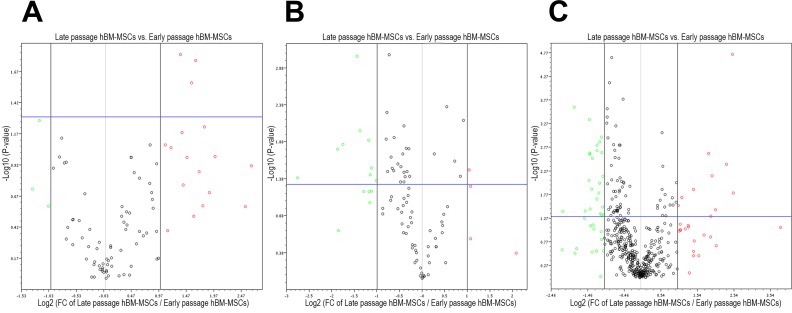
Volcano plots of human mesenchymal stem cell related gene A., cellular senescence related gene B. and miRNA C. expression changes in late passage *versus* early passage hBM-MSCs Red dots are the genes whose expression increased more than 2 fold, while green dots are decreased more than 2 fold. Vertical grey side-lines represent fold-change cutoff (≥2 fold) and horizontal blue line represents *p*-value cutoff (*P* < 0.05). Genes and miRNAs whose fold expression changes and *P*-values exceeded the boundaries are listed in the Table 1.

To further evaluate the hBM-MSCs, we measured the expression of 162 different genes related to stemness, mesenchymal stem cells and cell senescence using commercial qPCR arrays at P3-P4 and P6-P7. Altogether, the expression of 154 genes was detected (C_t_ < 33) in early passage MSCs and the expression of 156 genes was detected in late passage MSCs. From 162 genes, 4 genes were significantly (*P* < 0.05) up-regulated (≥2 fold) and 9 genes were significantly down-regulated in late passage hBM-MSCs when compared with early passage MSCs (Figure 5A-5B, Table 1). This represents 8.02% of all genes investigated in the study. In order to better understand the underlying biological processes in late passage BM-MSCs, we performed gene enrichment analysis of set of 13 genes with significantly altered expression (Table 2).

**Table 1:** Significant fold regulation of genes and miRNAs in late passage versus early passage hBM-MSCs

Gene/miRNA Symbol	Fold Regulation	*P*-value	miRNA Symbol	Fold Regulation	*P*-value
*ACTA2*	3.1249	0.017625	*hsa-miR-935*	−4.4097	0.038848
*POU5F1*	2.9597	0.026534	*hsa-miR-193a-3p*	−3.5535	0.000240
*PTPRC*	2.5796	0.015727	*hsa-miR-200a-3p*	−2.8797	0.038458
*THBS1*	2.0582	0.031849	*hsa-miR-187-3p*	−2.6816	0.008664
*E2F3*	−6.7673	0.040950	*hsa-miR-192-5p*	−2.6692	0.000444
*CCNB1*	−3.6560	0.016672	*hsa-miR-130b-5p*	−2.6437	0.002407
*CHEK1*	−3.3642	0.014370	*hsa-miR-218-5p*	−2.6433	0.002247
*PLAU*	−2.7200	0.000930	*hsa-miR-92a-1-5p*	−2.6194	0.034756
*TBX2*	−2.5941	0.009478	*hsa-miR-877-5p*	−2.5503	0.012980
*TBX3*	−2.2662	0.012653	*hsa-miR-337-3p*	−2.5181	0.002904
*CDC25C*	−2.2195	0.037200	*hsa-miR-106b-3p*	−2.3068	0.021249
*E2F1*	−2.1738	0.030206	*hsa-miR-139-5p*	−2.3091	0.001997
*PCNA*	−2.0236	0.044944	*hsa-miR-455-5p*	−2.2997	0.001550
*hsa-miR-422a*	5.7364	0.016040	*hsa-miR-188-3p*	−2.2305	0.015998
*hsa-miR-376b-3p*	5.6578	0.000018	*hsa-miR-875-3p*	−2.1947	0.009676
*hsa-miR-200a-5p*	5.0577	0.003929	*hsa-miR-224-5p*	−2.1874	0.001306
*hsa-miR-219a-2-3p*	4.0963	0.036284	*hsa-miR-29a-5p*	−2.1573	0.043583
*hsa-miR-639*	3.8552	0.006863	*hsa-miR-25-5p*	−2.1203	0.046255
*hsa-miR-223-3p*	3.7575	0.049123	*hsa-miR-660-5p*	−2.1088	0.001035
*hsa-miR-608*	3.6014	0.002329	*hsa-miR-576-5p*	−2.0967	0.002239
*hsa-miR-429*	2.7041	0.013235	*hsa-miR-15b-3p*	−2.0742	0.031753
*has-miR-210-3p*	2.1681	0.019652	*hsa-miR-29b-1-5p*	−2.0528	0.006955
*hsa-miR-335-5p*	2.0328	0.028915	*hsa-miR-7-5p*	−2.0189	0.045330

**Table 2:** Gene Onthology (GO) term* enrichment analysis of set of 13 genes with altered expression in late passage hBM-MSCs

Gene Onthology category	Biological process	Sample frequency	Background frequency	*P*-value
GO:0050896	response to stimulus	13	7510	1.34E-02
GO:0060255	regulation of macromolecule metabolic process	12	5396	6.93E-03
GO:0051716	cellular response to stimulus	12	6121	3.01E-02
GO:0006950	response to stress	11	3492	1.26E-03
GO:0009893	positive regulation of metabolic process	10	3384	1.74E-02
GO:0009059	macromolecule biosynthetic process	10	3614	3.24E-02
GO:0010604	positive regulation of macromolecule metabolic process	10	2579	1.30E-03
GO:0048513	organ development	9	2728	3.76E-02
GO:0031325	positive regulation of cellular metabolic process	9	2750	4.03E-02
GO:0042127	regulation of cell proliferation	9	1438	1.51E-04
GO:0033554	cellular response to stress	8	1630	9.66E-03
GO:0007049	cell cycle	8	1290	1.61E-03
	**Cellular function**			
GO:0005667	transcription factor complex	4	291	2.82E-02

* Gene Ontology (GO) terms were used to define the biological processes, cellular and molecular functions using the Gene Ontology Consortium. Background frequency is the number of genes annotated to a GO term in the entire *H*. sapiens background set, while sample frequency is the number of genes annotated to that GO term in the input list. The terms listed in the table are the most frequently annotated with *P* < 0.05. No genes were statistically significantly annotated to GO terms for molecular functions.

### miRNA expression

The miRNA profiles of hBM-MSCs from early and late passages were analyzed with the commercial qPCR-based array for human miRNA. Overall, the expression of 358 miRNAs was detected (C_t_ < 33) in early passage hBM-MSCs and the expression of 365 miRNAs was detected in late passage cells. Analysis showed significant (*P* < 0.05) ≥2 fold changes in expression of 33 of 420 miRNAs (Figure 5C, Table 1), and these constituted 7.86% of all evaluated miRNAs.

## DISCUSSION

In this study we isolated MSCs from residual human bone marrow transplantation material, as described earlier [28], expanded *in vitro* to clinically relevant numbers and characterized these cells by evaluating adherence to plastic, morphology, proliferative capacity, immunophenotype, senescence status, karyotype stability, gene and miRNA expression profiling, as proposed by ISCT [29], EMA [30], and FDA [31]. hBM-MSC lifespan was categorized as early passage (P3-P4) and late passage (P6-P7) according to proliferation ability and the percentage of SA-β-gal, similarly as proposed before [32].

Proliferation is a fundamental property of stem cells necessary for self-renewal and expansion and defining stem cell degree of stemness [33]. Population doubling (PD) is a precise way to measure cell growth [34] and is recommended by the Cell Products Working Party (EMA) to describe the time for cells in culture [35]. We showed that hBM-MSCs at early passages are highly proliferative. Cryopreserved BM-MSCs at P1 can be expanded to high clinically relevant yield of cells within two weeks at P3-P4 with CPDs 8.08±0.74 (Figure 1D), which would result in hundreds of millions of cells. MSCs at early passages maintained a spindle-shaped or fibroblast-like morphology (Figure 1A), typical for adult hBM-MSCs [36], were adherent and exhibited immunophenotype (Figure 3A-3C) in accordance with ISCT guidelines [29].

Genomic instability of MSCs is one of important concern for clinical use of MSCs [37] because it enables the cells to acquire tumor cell characteristics [11]. Therefore the cytogenetic analysis is essential for verifying the safety of MSCs [38] since the maintenance of a normal karyotype is a reliable indicator of genetic stability of MSCs [39]. By conventional karyotyping of cultured hBM-MSCs using G-banding, which is still a gold standard of all cytogenetic techniques [40], we showed that BM-MSCs at P3-P4 had a normal karyotype and none of the samples had clonal aberrations (Figure 4A). These results indicate that the genomic stability of our MSCs would not prevent their potential use in a clinical application, similarly as shown earlier [15].

By expanding hBM-MSCs using additional three passages (until P6-P7) we investigated the possibility to achieve additional clinically relevant amounts of cells. However, in the late passages the hBM-MSCs growth gradually decreased (Figure 1D) and cells acquired an enlarged flattened morphology (Figure 1B-1C), indicating MSC senescence [41]. Cellular senescence is defined as irreversible cell cycle arrest [17]. By staining cells for SA-β-galactosidase, the most widely used biomarker for senescent cells [42], we confirmed that after 15.08±3.04 PDs (Figure 1D) almost half of late passage hBM-MSCs reached senescence, whereas only a few cells stained positively in the early passages (Figure 3A-3B).

Interestingly, the onset of senescence in long-term culture manifested differently on hBM-MSC immunophenotype in each sample. Surface marker expression of #1 sample remained stable throughout the *in vitro* expansion (Figure 2D), in agreement with Dmitrieva et al. [43] and Somasundaram et al. studies [44]. Dmitrieva et al. demonstrated that hBM-MSC enter senescence after P3-P4, but the cells were CD105/CD90/CD166/CD73 positive and negative for CD34, CD19, CD14 and CD45 at all passages. Somasundaram et al. revealed remarkable (>90%) expression of CD73, CD90, and CD105 and sparse (< 10%) expression of CD34, CD45, and HLA-DR of hBM-MSC irrespective of extensive culturing when the majority of samples lost potential to grow beyond P15. However, the expression of negative markers increased up to 5.10% in #2 sample in P7, although expression of positive markers remained stable (Figure 2E). Moreover, part of non-proliferating MSC population of #3 sample lost the expression of positive markers and gained the expression of negative markers in P7 (Figure 2F). Wagner et al. [45] has demonstrated that *in vitro* expansion has a major impact on the level of surface marker expression of human BM-MSCs. Surface antigen detection was much higher in early passages when compared to senescent passages. However, quantification (%) of expression was not presented in that study. Our results were unexpected and indicate that identification of late passage senescent MSCs by using cell-surface markers can be complicated. Therefore possible changes in standard surface marker expression during prolonged *in vitro* expansion require further investigations. We also revealed long-term culture-related, however not statistically significant, differences in cell granularity, another hBM-MSC senescence associated feature [46]. Interestingly, the karyotype of late passage senescent cells remained stable (Figure 4B), compatible with data obtained on long-term expanded hBM-MSCs by other groups [5, 14] and opposing to the recent finding that senescence-prone human MSCs are highly aneuploid [47].

To date no molecular markers are available, which specifically reflect the degree of cellular aging in a population of MSCs [48]. Molecular analysis of a suitable panel of genes might provide a powerful tool to track cellular aging of MSCs and thus to assess efficiency and safety of long-term expansion [42]. Real-time quantitative PCR is the gold-standard technique for gene expression measurements [49], therefore we investigated the cells using qPCR arrays. Transcriptome analysis of 162 different genes revealed 4 significantly (*P* < 0.05) up-regulated (≥2 fold) genes and 9 significantly down-regulated genes in P6-P7 hBM-MSCs when compared to P3-P4 cells (Table 1). *Pou5f1* (Oct4) is a critical regulator of pluripotency in embryonic stem cells and might be reactivated in response to culture conditions [50]. Exogenous OCT4 overexpression has been shown to induce early senescence of hBM-MSCs [51], virtually consistently with our observations. *PTPRC* encodes the protein tyrosine phosphatase CD45 not characteristic for hMSCs [29] and its overexpression decreases cytokine-induced signaling [52]. *ACTA2*, which was the most upregulated in our study, codes a smooth muscle α actin isoform enabling hBM-MSCs to contract the extracellular matrix (ECM) components [53]. *THBS1* codes thrombospondin-1, which is secreted and incorporated into ECM [54]. We determined *THBS1* upregulation in senescent hBM-MSCs in concordance with Yoo et al. report [55]. *PLAU* gene encodes enzyme urokinase-type plasminogen activator (uPA), which regulates ECM degradation, cell adhesion, and inflammatory cell activation [56] and which activity depends on cytoskeleton reorganization [57]. An impairment of cytoskeleton remodeling and/or organization has been associated with hBM-MSC senescence [58]. E2F1 and E2F3 control the expression of numerous genes involved in DNA replication and cell cycle progression. Deregulation of these transcription factors results in the induction of senescence [59], with the loss of E2F3, which was the most downregulated in this study, having the most pronounced effect [60]. *TBX2* and *TBX3* encode T-box proteins that function as transcriptional repressors [61]. Inhibition of both leads to cell senescence [62]. In contrast to our findings, Choi et al. showed higher TBX2 expression in late passage senescent hBM-MSCs [63]. Chk1 protein kinase is essential for the human G2 DNA damage checkpoint [64] and has been shown to be downregulated in senescent hBM-MSC [65]. *PCNA* codes proliferating cell nuclear antigen expressed exclusively in actively proliferating cells [66]. E2F1-3 induces expression of PCNA [67], which is regulated by Chk1 [68]. We showed *PCNA* repression in late passage senescent hBM-MSCs, in compliance with Choi et al. report [63]. Human Cdc25C phosphatase is a key activator of the cyclin B1/Cdk1 complex [69], which is essential for entry into mitosis [70]. CDC25C inhibition promotes cell cycle arrest [71], and G2/M arrest is characteristic for stress-induced premature senescence [72]. We showed *CCNB1* downregulation in senescent hBM-MSCs, in agreement with Noh et al. study [65]. Functional gene ontology analysis revealed that these genes are associated with biological processes as cell cycle, metabolism, cell aging, and response to stress (Table 2), all of which are important causes of cellular senescence [73]. In sum, these results together with literature data strongly suggest that identified 13 genes are interconnectedly related to hBM-MSC premature senescence. On the other hand, to our knowledge, we for the first time show that the expression of *POU5F1, PTPRC, ACTA2, E2F1, E2F3, Tbx3, PLAU* and *CDC25C* genes is altered in senescent hBM-MSCs during long-term expansion *in vitro*.

PCR array data were deposited into a public database Gene Expression Omnibus (http://www.ncbi.nlm.nih.gov/geo/) under accession number GSE68933.

The p53/p21/Rb and p16/RB axes are key signaling pathways involved in the induction of cell senescence [74]. In particular, RB and its family members, p107 and p130, are essential for the onset of senescence cell cycle arrest [17]. An unexpected finding of this study was the constant (within 2 fold) expression and/or nonsignificant changes (*P*≥0.05) of the expression of these crucial genes (data available in the database GEO under accession number GSE68933). These results stand out from contrary data obtained by other laboratories. Cheng et al. demonstrated that *p16*, *p21*, and *p53* are significantly upregulated in senescent hBM-MSCs [75]. Shibata et al. showed significant increase in the expression level of *p16* and no significant changes in the expression of *p21* and *p53* at the end of hBM-MSC life span [76], similar results are reported by Tarte with colleagues [16]. Kim et al. showed unaltered *p16* expression and reduced expression of *p53* during long-term culturing of hBM-MSCs [14]. However, we cannot rule out the possibility that genes exhibiting a less than twofold change may be of biologic value [77]. While the functions of RB1, p107 and p130 in the biology of MSCs remain largely uncharacterized [78].

MicroRNAs, also called miRNAs, are small 19-22 nucleotide sequences of noncoding RNA that work as endogenous epigenetic key gene expression regulators [79]. Only recently senescence-associated miRNAs (SA-miRNAs) have emerged as important effectors of senescence [80]. Therefore we were particularly interested in the possible involvement of the miRNAs in the regulation of hBM-MSC senescence. By using miRNA qPCR array, we identified 33 miRNAs with altered expression in late passage senescent hBM-MSCs (Table 1). Among the top downregulated, *miR-935* previously has been shown to be downregulated in elder hBM-MSCs [81]; *miR-193a* has been reported to regulate uPA [82], to target oxidative stress pathway [83], and not to be repressed in normal BM cells [84]. Additionally, *miR-337-5p* was shown to be differentially expressed in pediatric hBM-MSCs comparing to adult hBM-MSCs [85]. Yoo et al. demonstrated that *miR-29b* is downregulated in senescent hBM-MSC compared to young hBM-MSCs, but *miR-455-3p*, unlike in our study, was upregulated [86]. Among the most upregulated, *miR-376b* has been shown to be differentially expressed in pediatric hBM-MSCs when comparing to adult hBM-MSCs [85]; *miR-200a* has been associated with the oxidative stress [87] and shown to be activated in stress-induced senescent cells [88]. Tome et al. demonstrated *miR-335* increase in hBM-MSC *ex vivo* culture and correlation with cell senescence [89], similarly to our data. Together, these results along with other reports further firmly propose that hBM-MSCs underwent *in vitro* culture induced premature senescence. Besides, as far as we know, our report is the first to link the change of expression of new 30 miRNAs to hBM-MSC senescence during prolonged *in vitro* expansion.

Recently, Balakrishnan with colleagues determined that *miR-193a* and *miR-200a* of hBM-MSCs regulate hematopoietic stem cell niche-defining genes [90]. We demonstrated that the expression of these miRNAs, surprisingly, is one of the most altered in senescent hBM-MSCs (Table 1). Interestingly, senescence phenotype of the hematological patient hBM-MSCs only very lately has been revealed [91–96].

Taking everything into account, we state that MSCs isolated from residual bone marrow transplantation material and expanded to clinically relevant numbers are genomically stable and retain identity and high proliferation capacity. It is a crucial requisite for clinical application in terms of donor comfort and recipient safety. However, the cells enter senescence state after long-term expansion, most likely, due to culture-induced stress. We propose that the identified novel hBM-MSC senescence associated genes and miRNAs provide a better understanding of the mechanisms involved in hBM-MSC aging, significantly contributing to basic science and cellular therapy quality control development and revealing new clues of hematological disease processes for future investigations. Further larger research in this area is needed to validate the claims of this study.

## MATERIALS AND METHODS

### Bone marrow collection

Bone marrow (BM) specimens were collected from healthy adult donors after obtaining written informed consent at Vilnius University Santariskiu Clinic, Children Hematology and Transplantation Center. The study was reviewed by Vilnius Regional Committee of Biomedical Research, Lithuania (Permission No 158200-09-381-104).

### Isolation of MSCs

MSCs were isolated from 3 donors (#1 female, age 24; #2 male, age 38; #3 female, age 28) using red blood cell lysis method as described earlier [28]. Briefly, BM samples were mixed with erythrocyte lysis buffer (Qiagen, Germany) and centrifuged for 5 min at 480g. After removal of the supernatant, the pellet was resuspended with 5 ml of RPMI 1640 medium (Invitrogen, UK) and washed twice through centrifugation. Finally, all amount of resuspended cell suspension was placed into T75 cm^2^ tissue culture flask (BD Biosciences, France) and allowed to adhere for 24 hours in the DMEM medium containing 10% of fetal bovine serum (FBS) (StemCell Technologies, Canada) and 1% penicillin/streptomycin (Gibco, USA) at 37°C with 5% CO_2_ and fully humidified atmosphere.

### Culture of MSCs

After 24 hours the medium was removed and the cells were washed with phosphate buffered saline (PBS). Human MesenCult MSC Basal Medium containing 10% of MesenCult FBS for human MSCs (StemCell Technologies, Canada) and 1% penicillin/streptomycin (Gibco, USA) was used for subsequent cultivation of MSCs. The medium was changed every 3-4 days. When cells reached 80-90% confluence, they were harvested with 0.25% trypsin-EDTA (Invitrogen, UK), counted and subcultured with seeding density 4000/cm^2^ into new T75 cm2 flasks under the standard conditions.

### Morphology

Cell morphology was determined using Nicon inverted phase contrast microscope (models Eclipse T*i*-S and TS100) and NIS-Elements imaging software (version 3.22.00). For spread cell area analysis nineteen cells from each image were chosen at random and manually outlined. Individual cell areas were measured. Image analysis was performed in ImageJ v1.50e image processing tool set.

### Cryopreservation and thawing

MSCs were cryopreserved at P1 and P3-P4. Cells were mixed with MSC Freezing Solution (Biological Industries, Israel) and placed into Mr. Frosty Freezing Container (Thermo Scientific, USA) in −80°C freezer for −1°C/minute freezing rate. After 24 hours cryovials were transferred to −150°C freezer for storage. After six months of storing samples were rapidly thawed by placing cryovials in a water bath at 37°C and diluted in a slow dropwise manner with pre-warmed fresh culture medium. After centrifugation at 150 g for 10 min, MSCs were plated with seeding density 4000/cm^2^ into T75 cm^2^ flasks and incubated under the standard conditions.

### Cell number and proliferation kinetics

Cell number was determined using a CASY cell counter and analyzer (Roche, Germany) at each passage and long-term growth kinetics *in vitro* was assessed by determining cumulative population doublings (CPDs). The number of population doublings (PDs) was calculated using the formula: PD = log(X_b_/X_a_)·3.3, where X_a_ represents the initial cell number, X_b_ represents the cell harvest number, and 3.3 is a coefficient. PD of each passage was calculated and added to the PD of the previous passage level to obtain the CPD.

### Flow cytometry

hBM-MSCs were characterized at P3-P4 and P6-P7 by flow cytometry using antibodies to CD44, CD73, CD90, and CD105 cell surface markers and using a mixture of antibodies to CD34, CD11b, CD19, CD45, HLA-DR cell surface markers (BD Stemflow™ Human MSC Analysis Kit). After harvesting, the cells were washed with PBS and treated according to the manufacturer's protocol. Viability of the MSCs samples was assessed by 5-minute 7-AAD staining and cell granularity was determined by side-scattered (SSC) light evaluation. Cytometric measurements were performed on BD LSR II flow cytometer. 10.000 cells per tube were analyzed with FlowJo X software.

### Senescence-associated β-galactosidase staining

Senescence of cultivated hBM-MSCs at passages 3-4 and 6-7 was studied using Senescence Cells Histochemical Staining Kit (Sigma-Aldrich, Germany) according to the manufacturer's protocol. At the end of staining procedure, ten pictures were taken from random areas of each culture. The percentage of senescent cells was calculated using the following formula: (number of cells with intracellular blue deposits/total number of cells) x 100%.

### Karyotype analysis

Cytogenetic evaluation by G-banding method was conducted on hBM-MSCs at passages 3-4 and 6-7. Colchicine was added into each culture at a final concentration of 0.2 μg/ml for 4 hours at 37°C. MSCs were harvested using trypsin and resuspended in a hypotonic 0.075 mM KCl solution for 30 min at 37°C. After centrifugation the cells were fixed with methanol:acetic acid (3:1) solution. After dropping the cell suspension onto microscope slides, these were trypsinized and stained with Giemsa solution (Sigma-Aldrich, Germany). Slides were scanned, metaphases we captured and analyzed with Leica CytoVision® (USA) platform. 7 to 17 metaphase spreads were analyzed for chromosome number and structure abnormalities at each established passage. Karyotypes were described following the recommendations of the International System for Human Cytogenetic Nomenclature 2013 [97].

### RNA isolation

Total RNA was isolated from hBM-MSCs using miRNeasy Mini Kit (Qiagen, Germany) following the manufacturer's instructions. Total RNA concentration and quality were checked using a NanoDrop spectrophotometer and verified by analysis on an Agilent 2100 Bioanalyzer using RNA 6000 Nano LabChip (Agilent Technologies, USA).

### PCR arrays

hBM-MSC samples of P3-P4 and P6-P7 were analyzed using Human Mesenchymal Stem Cell RT^2^ Profiler™ PCR Array (PAHS-082Z, SABiosciences, Qiagen) and Human Cellular Senescence RT^2^ Profiler™ PCR Array (PAHS-050Z). Template cDNA was synthesized from 800 ng of the total RNA using the RT^2^ First Strand Kit (Qiagen) following manufacturer's protocol. The reaction mix was prepared by mixing cDNA with 2x RT^2^ SYBR Green ROX FAST Mastermix (Qiagen) and 20 ml of the cocktail was aliquoted into each well on the PCR array. Each array consisted of a panel of 96 primer sets of 84 mesenchymal stem cell or cellular senescence genes, 5 housekeeping genes, and 7 quality controls. PCR arrays were performed in Rotor-Gene Q thermocycler (Qiagen), as follows: 95°C, 10 sec for initial denaturation, and 40 cycles of 95°C for 15 sec and 60°C for 30 sec. Each sample was tested in technical duplicate. The data were analyzed using web-based RT^2^ Profiler PCR Array Data Analysis v3.5 software. The fold-change in target gene expression was calculated using the ΔΔC_t_ method and normalized to the geometric mean of 5 housekeeping genes (ACTB, B2M, GAPDH, HPRT1, and RPLP0) according to SABioscience guide. A more than two-fold change in gene expression was considered as the up- or down-regulation of a specific gene expression. Differences were considered significant when *P* value < 0.05.

### miRNA PCR array

The miRNA levels in hBM-MSCs of early and late passages were analyzed with miRNome miScript miRNA PCR Array (MIHS-216ZR-4, SABiosciences, Qiagen). Template cDNA was synthesized from 600 ng of the total RNA with miScript II RT Kit using miScript HiSpec Buffer (Qiagen) following manufacturer's protocol. The templates were mixed with RT^2^ SYBR Green qPCR Master Mix (Qiagen) and 20 μl aliquoted into each well of 5 PCR arrays. Each array consisted of a panel of 96 primer sets of 84 miRNAs of interest, 2 *C*. *elegans* miR-39, and 10 controls. PCR was performed in Rotor-Gene Q thermocycler (Qiagen), as follows: 15 min at 95°C and 40 cycles of 15 sec at 94°C, 30 sec at 55°C, and 30 sec at 70°C. Each sample was tested in technical duplicate. The miRNA data were analyzed using online software miScript miRNA PCR Array Data Analysis. The relative expression of each target miRNA was determined with the ΔΔCt method and normalized to the geometric mean of 6 small RNAs (SNORD61, SNORD68, SNORD72, SNORD95, SNORD96A, and RNU6-2) according to SABioscience guide. A miRNA was considered differentially expressed if it showed more than two-fold change and *P* value < 0.05 indicated significance.

### Gene ontology analysis

Gene Ontology Consortium (http://geneontology.org/) was used for enrichment analysis of specific gene sets [98]. Genes were classified to gene ontology (GO) terms in three categories: molecular function, cellular component and biological process.

### Data analysis

Statistical analysis was performed using SPSS software (version 21). The Student's paired t-test was performed to assess statistical differences which were considered significant when *P* value < 0.05.

### Data access

PCR array data were deposited into a public database Gene Expression Omnibus (http://www.ncbi.nlm.nih.gov/geo/) under accession number GSE68933.

## References

[1] Tomar GB, Srivastava RK, Gupta N, Barhanpurkar AP, Pote ST, Jhaveri HM, Mishra GC, Wani MR (2010). Human gingiva-derived mesenchymal stem cells are superior to bone marrow-derived mesenchymal stem cells for cell therapy in regenerative medicine. Biochem Biophys Res Commun.

[2] Nombela-Arrieta C, Ritz J, Silberstein LE (2011). The elusive nature and function of mesenchymal stem cells. Nat Rev Mol Cell Biol.

[3] Wei X, Yang X, Han ZP, Qu FF, Shao L, Shi YF (2013). Mesenchymal stem cells: a new trend for cell therapy. Acta Pharmacol Sin.

[4] Roselli EA, Lazzati S, Iseppon F, Manganini M, Marcato L, Gariboldi MB, Maggi F, Grati FR, Simoni G (2013). Fetal mesenchymal stromal cells from cryopreserved human chorionic villi: cytogenetic and molecular analysis of genome stability in long-term cultures. Cytotherapy.

[5] Redaelli S, Bentivegna A, Foudah D, Miloso M, Redondo J, Riva G, Baronchelli S, Dalpra L, Tredici G (2012). From cytogenomic to epigenomic profiles: monitoring the biologic behavior of *in vitro* cultured human bone marrow mesenchymal stem cells. Stem Cell Res Ther.

[6] Wernicke CM, Grunewald TG, Juenger H, Kuci S, Kuci Z, Koehl U, Mueller I, Doering M, Peters C, Lawitschka A, Kolb HJ, Bader P, Burdach S, von Luettichau I (2011). Mesenchymal stromal cells for treatment of steroid-refractory GvHD: a review of the literature and two pediatric cases. Int Arch Med.

[7] Bongso A, Fong CY (2013). The therapeutic potential, challenges and future clinical directions of stem cells from the Wharton's jelly of the human umbilical cord. Stem Cell Rev.

[8] Pikula M, Marek-Trzonkowska N, Wardowska A, Renkielska A, Trzonkowski P (2013). Adipose tissue-derived stem cells in clinical applications. Expert Opin Biol Ther.

[9] Ding DC, Chang YH, Shyu WC, Lin SZ (2015). Human umbilical cord mesenchymal stem cells: a new era for stem cell therapy. Cell Transplant.

[10] Wang Y, Han ZB, Song YP, Han ZC (2012). Safety of mesenchymal stem cells for clinical application. Stem Cells Int.

[11] Hanahan D, Weinberg RA (2011). Hallmarks of cancer: the next generation. Cell.

[12] Kim SY, Im K, Park SN, Kwon J, Kim JA, Choi Q, Hwang SM, Han SH, Kwon S, Oh IH, Lee DS (2015). Asymmetric aneuploidy in mesenchymal stromal cells detected by in situ karyotyping and fluorescence in situ hybridization: suggestions for reference values for stem cells. Stem Cells Dev.

[13] Wang Y, Huso DL, Harrington J, Kellner J, Jeong DK, Turney J, McNiece IK (2005). Outgrowth of a transformed cell population derived from normal human BM mesenchymal stem cell culture. Cytotherapy.

[14] Kim J, Kang JW, Park JH, Choi Y, Choi KS, Park KD, Baek DH, Seong SK, Min HK, Kim HS (2009). Biological characterization of long-term cultured human mesenchymal stem cells. Arch Pharm Res.

[15] Jones M, Varella-Garcia M, Skokan M, Bryce S, Schowinsky J, Peters R, Vang B, Brecheisen M, Startz T, Frank N, Nankervis B (2013). Genetic stability of bone marrow-derived human mesenchymal stromal cells in the Quantum System. Cytotherapy.

[16] Tarte K, Gaillard J, Lataillade J-J, Fouillard L, Becker M, Mossafa H, Tchirkov A, Rouard H, Henry C, Splingard M, Dulong J, Monnier D, Gourmelon P, Gorin N-C, Sensebé L (2010). Clinical-grade production of human mesenchymal stromal cells: occurrence of aneuploidy without transformation. Blood.

[17] Ohtani N, Hara E (2013). Roles and mechanisms of cellular senescence in regulation of tissue homeostasis. Cancer Sci.

[18] Shvarts A, Brummelkamp TR, Scheeren F, Koh E, Daley GQ, Spits H, Bernards R (2002). A senescence rescue screen identifies BCL6 as an inhibitor of anti-proliferative p19(ARF)-p53 signaling. Genes Dev.

[19] Sepulveda JC, Tome M, Fernandez ME, Delgado M, Campisi J, Bernad A, Gonzalez MA (2014). Cell senescence abrogates the therapeutic potential of human mesenchymal stem cells in the lethal endotoxemia model. Stem Cells.

[20] Shay JW, Roninson IB (2004). Hallmarks of senescence in carcinogenesis and cancer therapy. Oncogene.

[21] Gosselin K, Martien S, Pourtier A, Vercamer C, Ostoich P, Morat L, Sabatier L, Duprez L, T'Kint de Roodenbeke C, Gilson E, Malaquin N, Wernert N, Slijepcevic P, Ashtari M, Chelli F, Deruy E (2009). Senescence-associated oxidative DNA damage promotes the generation of neoplastic cells. Cancer Res.

[22] Rubio D, Garcia-Castro J, Martin MC, de la Fuente R, Cigudosa JC, Lloyd AC, Bernad A (2005). Spontaneous human adult stem cell transformation. Cancer Res.

[23] Rosland GV, Svendsen A, Torsvik A, Sobala E, McCormack E, Immervoll H, Mysliwietz J, Tonn JC, Goldbrunner R, Lonning PE, Bjerkvig R, Schichor C (2009). Long-term cultures of bone marrow-derived human mesenchymal stem cells frequently undergo spontaneous malignant transformation. Cancer Res.

[24] de la Fuente R, Bernad A, Garcia-Castro J, Martin MC, Cigudosa JC (2010). Retraction: Spontaneous human adult stem cell transformation. Cancer Res.

[25] Torsvik A, Rosland GV, Svendsen A, Molven A, Immervoll H, McCormack E, Lonning PE, Primon M, Sobala E, Tonn JC, Goldbrunner R, Schichor C, Mysliwietz J, Lah TT, Motaln H, Knappskog S (2010). Spontaneous malignant transformation of human mesenchymal stem cells reflects cross-contamination: putting the research field on track - letter. Cancer Res.

[26] Ben-David U, Mayshar Y, Benvenisty N (2011). Large-scale analysis reveals acquisition of lineage-specific chromosomal aberrations in human adult stem cells. Cell Stem Cell.

[27] Sensebe L, Tarte K, Galipeau J, Krampera M, Martin I, Phinney DG, Shi Y (2012). Limited acquisition of chromosomal aberrations in human adult mesenchymal stromal cells. Cell Stem Cell.

[28] Gudleviciene Z, Kundrotas G, Liudkeviciene R, Rascon J, Jurga M (2015). Quick and effective method of bone marrow mesenchymal stem cell extraction. Open Medicine.

[29] Dominici M, Le Blanc K, Mueller I, Slaper-Cortenbach I, Marini F, Krause D, Deans R, Keating A, Prockop D, Horwitz E (2006). Minimal criteria for defining multipotent mesenchymal stromal cells. The International Society for Cellular Therapy position statement. Cytotherapy.

[30] EMA (2011). Reflection paper on stem cell-based medicinal products.

[31] Bailey AM (2012). Balancing tissue and tumor formation in regenerative medicine. Sci Transl Med.

[32] Stenderup K, Justesen J, Clausen C, Kassem M (2003). Aging is associated with decreased maximal life span and accelerated senescence of bone marrow stromal cells. Bone.

[33] Rich IN, Rich IN (2015). Measurement of Hematopoietic Stem Cell Proliferation, Self-Renewal, and Expansion Potential. Stem Cell Protocols.

[34] Greenwood SK, Hill RB, Sun JT, Armstrong MJ, Johnson TE, Gara JP, Galloway SM (2004). Population doubling: a simple and more accurate estimation of cell growth suppression in the *in vitro* assay for chromosomal aberrations that reduces irrelevant positive results. Environ Mol Mutagen.

[35] Barkholt L, Flory E, Jekerle V, Lucas-Samuel S, Ahnert P, Bisset L, Buscher D, Fibbe W, Foussat A, Kwa M, Lantz O, Maciulaitis R, Palomaki T, Schneider CK, Sensebe L, Tachdjian G (2013). Risk of tumorigenicity in mesenchymal stromal cell-based therapies—bridging scientific observations and regulatory viewpoints. Cytotherapy.

[36] Campagnoli C, Roberts IA, Kumar S, Bennett PR, Bellantuono I, Fisk NM (2001). Identification of mesenchymal stem/progenitor cells in human first-trimester fetal blood, liver, and bone marrow. Blood.

[37] Sensebe L (2013). Beyond genetic stability of mesenchymal stromal cells. Cytotherapy.

[38] Hwang SM, See CJ, Choi J, Kim SY, Choi Q, Kim JA, Kwon J, Park SN, Im K, Oh IH, Lee DS (2013). The application of an in situ karyotyping technique for mesenchymal stromal cells: a validation and comparison study with classical G-banding. Exp Mol Med.

[39] Borgonovo T, Vaz IM, Senegaglia AC, Rebelatto CL, Brofman PR (2014). Genetic evaluation of mesenchymal stem cells by G-banded karyotyping in a Cell Technology Center. Rev Bras Hematol Hemoter.

[40] Liehr T, Pellestor F, Liehr T (2009). Molecular Cytogenetics: The Standard FISH and PRINS Procedure. Fluorescence In Situ Hybridization (FISH) — Application Guide.

[41] Sethe S, Scutt A, Stolzing A (2006). Aging of mesenchymal stem cells. Ageing Res Rev.

[42] Wagner W, Bork S, Lepperdinger G, Joussen S, Ma N, Strunk D, Koch C (2010). How to track cellular aging of mesenchymal stromal cells?. Aging (Albany NY).

[43] Dmitrieva RI, Minullina IR, Bilibina AA, Tarasova OV, Anisimov SV, Zaritskey AY (2012). Bone marrow- and subcutaneous adipose tissue-derived mesenchymal stem cells: differences and similarities. Cell Cycle.

[44] Somasundaram I, Mishra R, Radhakrishnan H, Sankaran R, Garikipati VN, Marappagounder D (2015). Human adult stem cells maintain a constant phenotype profile irrespective of their origin, Basal media, and long term cultures. Stem Cells Int.

[45] Wagner W, Horn P, Castoldi M, Diehlmann A, Bork S, Saffrich R, Benes V, Blake J, Pfister S, Eckstein V, Ho AD (2008). Replicative senescence of mesenchymal stem cells: a continuous and organized process. PLoS One.

[46] Schallmoser K, Bartmann C, Rohde E, Bork S, Guelly C, Obenauf AC, Reinisch A, Horn P, Ho AD, Strunk D, Wagner W (2010). Replicative senescence-associated gene expression changes in mesenchymal stromal cells are similar under different culture conditions. Haematologica.

[47] Estrada JC, Torres Y, Benguria A, Dopazo A, Roche E, Carrera-Quintanar L, Perez RA, Enriquez JA, Torres R, Ramirez JC, Samper E, Bernad A (2013). Human mesenchymal stem cell-replicative senescence and oxidative stress are closely linked to aneuploidy. Cell Death Dis.

[48] Laschober GT, Brunauer R, Jamnig A, Fehrer C, Greiderer B, Lepperdinger G (2009). Leptin receptor/CD295 is upregulated on primary human mesenchymal stem cells of advancing biological age and distinctly marks the subpopulation of dying cells. Exp Gerontol.

[49] Ballester M, Cordon R, Folch JM (2013). DAG expression: high-throughput gene expression analysis of real-time PCR data using standard curves for relative quantification. PLoS One.

[50] Lengner CJ, Welstead GG, Jaenisch R (2008). The pluripotency regulator Oct4: a role in somatic stem cells?. Cell Cycle.

[51] Jaganathan BG, Bonnet D (2012). Human mesenchymal stromal cells senesce with exogenous OCT4. Cytotherapy.

[52] Porcu M, Kleppe M, Gianfelici V, Geerdens E, De Keersmaecker K, Tartaglia M, Foa R, Soulier J, Cauwelier B, Uyttebroeck A, Macintyre E, Vandenberghe P, Asnafi V, Cools J (2012). Mutation of the receptor tyrosine phosphatase PTPRC (CD45) in T-cell acute lymphoblastic leukemia. Blood.

[53] Kinner B, Zaleskas JM, Spector M (2002). Regulation of smooth muscle actin expression and contraction in adult human mesenchymal stem cells. Exp Cell Res.

[54] Li SS, Ivanoff A, Bergstrom SE, Sandstrom A, Christensson B, van Nerven J, Holgersson J, Hauzenberger D, Arencibia I, Sundqvist KG (2002). T lymphocyte expression of thrombospondin-1 and adhesion to extracellular matrix components. Eur J Immunol.

[55] Yoo JK, Choi SJ, Kim JK (2013). Expression profiles of subtracted mRNAs during cellular senescence in human mesenchymal stem cells derived from bone marrow. Exp Gerontol.

[56] Begin P, Tremblay K, Daley D, Lemire M, Claveau S, Salesse C, Kacel S, Montpetit A, Becker A, Chan-Yeung M, Kozyrskyj AL, Hudson TJ, Laprise C (2007). Association of urokinase-type plasminogen activator with asthma and atopy. Am J Respir Crit Care Med.

[57] Irigoyen JP, Besser D, Nagamine Y (1997). Cytoskeleton reorganization induces the urokinase-type plasminogen activator gene via the Ras/extracellular signal-regulated kinase (ERK) signaling pathway. J Biol Chem.

[58] Madeira A, da Silva CL, dos Santos F, Camafeita E, Cabral JM, Sa-Correia I (2012). Human mesenchymal stem cell expression program upon extended ex-vivo cultivation, as revealed by 2-DE-based quantitative proteomics. PLoS One.

[59] Hong S, Paulson QX, Johnson DG (2008). E2F1 and E2F3 activate ATM through distinct mechanisms to promote E1A-induced apoptosis. Cell Cycle.

[60] Saavedra HI, Wu L, de Bruin A, Timmers C, Rosol TJ, Weinstein M, Robinson ML, Leone G (2002). Specificity of E2F1, E2F2, and E2F3 in mediating phenotypes induced by loss of Rb. Cell Growth Differ.

[61] Zirzow S, Ludtke TH, Brons JF, Petry M, Christoffels VM, Kispert A (2009). Expression and requirement of T-box transcription factors Tbx2 and Tbx3 during secondary palate development in the mouse. Dev Biol.

[62] Peres J, Davis E, Mowla S, Bennett DC, Li JA, Wansleben S, Prince S (2010). The Highly Homologous T-Box Transcription Factors, TBX2 and TBX3, Have Distinct Roles in the Oncogenic Process. Genes Cancer.

[63] Choi MR, In YH, Park J, Park T, Jung KH, Chai JC, Chung MK, Lee YS, Chai YG (2012). Genome-scale DNA methylation pattern profiling of human bone marrow mesenchymal stem cells in long-term culture. Exp Mol Med.

[64] Koniaras K, Cuddihy AR, Christopoulos H, Hogg A, O'Connell MJ (2001). Inhibition of Chk1-dependent G2 DNA damage checkpoint radiosensitizes p53 mutant human cells. Oncogene.

[65] Noh H, Ahn H-J, Lee W-J, Kwack K, Kwon Y (2010). The molecular signature of *in vitro* senescence in human mesenchymal stem cells. Genes Genom.

[66] Muinos-Lopez E, Rendal-Vazquez ME, Hermida-Gomez T, Fuentes-Boquete I, Diaz-Prado S, Blanco FJ (2012). Cryopreservation effect on proliferative and chondrogenic potential of human chondrocytes isolated from superficial and deep cartilage. Open Orthop J.

[67] Popov B, Petrov N (2014). pRb-E2F signaling in life of mesenchymal stem cells: Cell cycle, cell fate, and cell differentiation. Genes & Diseases.

[68] Zhang W, Qin Z, Zhang X, Xiao W (2011). Roles of sequential ubiquitination of PCNA in DNA-damage tolerance. FEBS Lett.

[69] Franckhauser C, Mamaeva D, Heron-Milhavet L, Fernandez A, Lamb NJ (2010). Distinct pools of cdc25C are phosphorylated on specific TP sites and differentially localized in human mitotic cells. PLoS One.

[70] Mitra J, Enders GH (2004). Cyclin A/Cdk2 complexes regulate activation of Cdk1 and Cdc25 phosphatases in human cells. Oncogene.

[71] Welford SM, Giaccia AJ (2011). Hypoxia and senescence: the impact of oxygenation on tumor suppression. Mol Cancer Res.

[72] Magimaidas A, Hoffman B, Liebermann D (2014). Abstract 2252: Gadd45b deficiency impairs G2/M cell cycle progression leading to premature senescence. Cancer Research.

[73] Kong Y, Cui H, Ramkumar C, Zhang H (2011). Regulation of senescence in cancer and aging. J Aging Res.

[74] Coleman PR, Hahn CN, Grimshaw M, Lu Y, Li X, Brautigan PJ, Beck K, Stocker R, Vadas MA, Gamble JR (2010). Stress-induced premature senescence mediated by a novel gene, SENEX, results in an anti-inflammatory phenotype in endothelial cells. Blood.

[75] Cheng H, Qiu L, Ma J, Zhang H, Cheng M, Li W, Zhao X, Liu K (2011). Replicative senescence of human bone marrow and umbilical cord derived mesenchymal stem cells and their differentiation to adipocytes and osteoblasts. Mol Biol Rep.

[76] Shibata KR, Aoyama T, Shima Y, Fukiage K, Otsuka S, Furu M, Kohno Y, Ito K, Fujibayashi S, Neo M, Nakayama T, Nakamura T, Toguchida J (2007). Expression of the p16INK4A gene is associated closely with senescence of human mesenchymal stem cells and is potentially silenced by DNA methylation during *in vitro* expansion. Stem Cells.

[77] Bellayr IH, Catalano JG, Lababidi S, Yang AX, Lo Surdo JL, Bauer SR, Puri RK (2014). Gene markers of cellular aging in human multipotent stromal cells in culture. Stem Cell Res Ther.

[78] Alessio N, Bohn W, Rauchberger V, Rizzolio F, Cipollaro M, Rosemann M, Irmler M, Beckers J, Giordano A, Galderisi U (2013). Silencing of RB1 but not of RB2/P130 induces cellular senescence and impairs the differentiation potential of human mesenchymal stem cells. Cell Mol Life Sci.

[79] Moreno-Moya JM, Vilella F, Simon C (2014). MicroRNA: key gene expression regulators. Fertil Steril.

[80] Bilsland AE, Revie J, Keith W (2013). MicroRNA and senescence: the senectome, integration and distributed control. Crit Rev Oncog.

[81] Pandey AC, Semon JA, Kaushal D, O'Sullivan RP, Glowacki J, Gimble JM, Bunnell BA (2011). MicroRNA profiling reveals age-dependent differential expression of nuclear factor kappaB and mitogen-activated protein kinase in adipose and bone marrow-derived human mesenchymal stem cells. Stem Cell Res Ther.

[82] Salvi A, Conde I, Abeni E, Arici B, Grossi I, Specchia C, Portolani N, Barlati S, De Petro G (2013). Effects of miR-193a and sorafenib on hepatocellular carcinoma cells. Mol Cancer.

[83] Deng H, Lv L, Li Y, Zhang C, Meng F, Pu Y, Xiao J, Qian L, Zhao W, Liu Q, Zhang D, Wang Y, Zhang H, He Y, Zhu J (2014). miR-193a-3p regulates the multi-drug resistance of bladder cancer by targeting the LOXL4 gene and the Oxidative Stress pathway. Mol Cancer.

[84] Gao XN, Lin J, Li YH, Gao L, Wang XR, Wang W, Kang HY, Yan GT, Wang LL, Yu L (2011). MicroRNA-193a represses c-kit expression and functions as a methylation-silenced tumor suppressor in acute myeloid leukemia. Oncogene.

[85] Candini O, Spano C, Murgia A, Grisendi G, Veronesi E, Piccinno MS, Ferracin M, Negrini M, Giacobbi F, Bambi F, Horwitz EM, Conte P, Paolucci P, Dominici M (2015). Mesenchymal progenitors aging highlights a miR-196 switch targeting HOXB7 as master regulator of proliferation and osteogenesis. Stem Cells.

[86] Yoo JK, Kim CH, Jung HY, Lee DR, Kim JK (2014). Discovery and characterization of miRNA during cellular senescence in bone marrow-derived human mesenchymal stem cells. Exp Gerontol.

[87] Mateescu B, Batista L, Cardon M, Gruosso T, de Feraudy Y, Mariani O, Nicolas A, Meyniel JP, Cottu P, Sastre-Garau X, Mechta-Grigoriou F (2011). miR-141 and miR-200a act on ovarian tumorigenesis by controlling oxidative stress response. Nat Med.

[88] Cufi S, Vazquez-Martin A, Oliveras-Ferraros C, Quirantes R, Segura-Carretero A, Micol V, Joven J, Bosch-Barrera J, Del Barco S, Martin-Castillo B, Vellon L, Menendez JA (2012). Metformin lowers the threshold for stress-induced senescence: a role for the microRNA-200 family and miR-205. Cell Cycle.

[89] Tome M, Sepulveda JC, Delgado M, Andrades JA, Campisi J, Gonzalez MA, Bernad A (2014). miR-335 correlates with senescence/aging in human mesenchymal stem cells and inhibits their therapeutic actions through inhibition of AP-1 activity. Stem Cells.

[90] Balakrishnan I, Yang X, Brown J, Ramakrishnan A, Torok-Storb B, Kabos P, Hesselberth JR, Pillai MM (2014). Genome-wide analysis of miRNA-mRNA interactions in marrow stromal cells. Stem Cells.

[91] Berenstein R, Blau O, Nogai A, Waechter M, Slonova E, Schmidt-Hieber M, Kunitz A, Pezzutto A, Doerken B, Blau IW (2015). Multiple myeloma cells alter the senescence phenotype of bone marrow mesenchymal stromal cells under participation of the DLK1-DIO3 genomic region. BMC Cancer.

[92] Andre T, Meuleman N, Stamatopoulos B, De Bruyn C, Pieters K, Bron D, Lagneaux L (2013). Evidences of early senescence in multiple myeloma bone marrow mesenchymal stromal cells. PLoS One.

[93] Ferrer RA, Wobus M, List C, Wehner R, Schonefeldt C, Brocard B, Mohr B, Rauner M, Schmitz M, Stiehler M, Ehninger G, Hofbauer LC, Bornhauser M, Platzbecker U (2013). Mesenchymal stromal cells from patients with myelodyplastic syndrome display distinct functional alterations that are modulated by lenalidomide. Haematologica.

[94] Geyh S, Oz S, Cadeddu RP, Frobel J, Bruckner B, Kundgen A, Fenk R, Bruns I, Zilkens C, Hermsen D, Gattermann N, Kobbe G, Germing U, Lyko F, Haas R, Schroeder T (2013). Insufficient stromal support in MDS results from molecular and functional deficits of mesenchymal stromal cells. Leukemia.

[95] Ding W, Secreto C, Wu X, Braggio E, Zhang Y, Smoley SA, Shanafelt TD, Davila J, Call TG, Van Dyke DL, Jelinek DF, Kay NE (2014). 3282 CLL Mesenchymal Stromal Cells Have Decreased Replicative Potential and Senescent Phenotype: Clinical and Biologic Implications.

[96] Zhao Y, Wu D, Fei C, Guo J, Gu S, Zhu Y, Xu F, Zhang Z, Wu L, Li X, Chang C (2015). Down-regulation of Dicer1 promotes cellular senescence and decreases the differentiation and stem cell-supporting capacities of mesenchymal stromal cells in patients with myelodysplastic syndrome. Haematologica.

[97] Shaffer LG, McGowan-Jordan J, Schmid M (2013). ISCN: an international system for human cytogenetic nomenclature.

[98] Ashburner M, Ball CA, Blake JA, Botstein D, Butler H, Cherry JM, Davis AP, Dolinski K, Dwight SS, Eppig JT, Harris MA, Hill DP, Issel-Tarver L, Kasarskis A, Lewis S, Matese JC (2000). Gene ontology: tool for the unification of biology. The Gene Ontology Consortium. Nat Genet.

